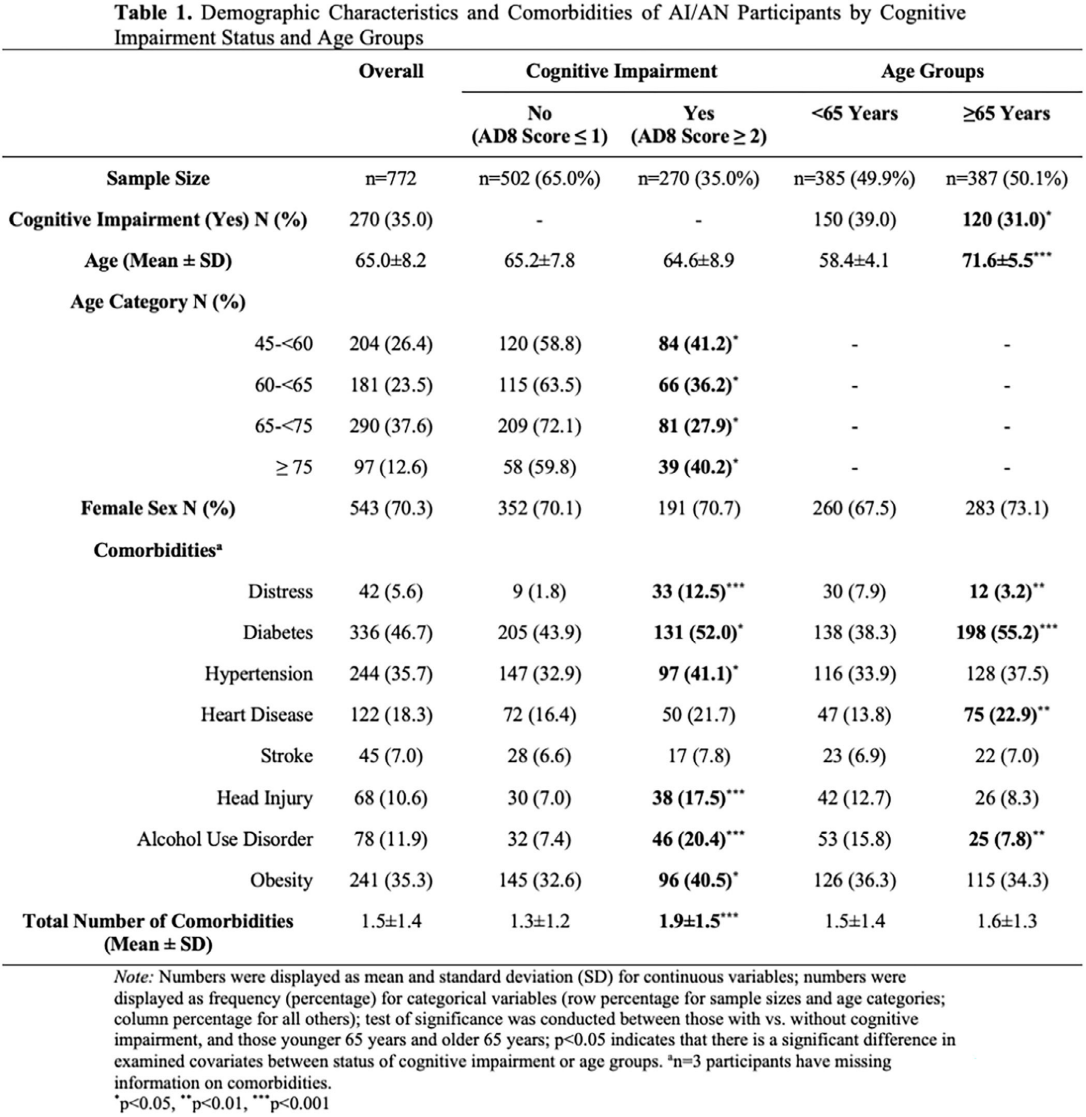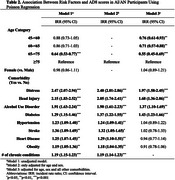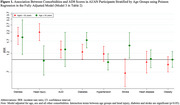# Cognitive Impairment and Its Associated Comorbidities in American Indian and Alaska Native Communities

**DOI:** 10.1002/alz70860_107680

**Published:** 2025-12-23

**Authors:** Wenjun Fan, Jiahui Dai, Yuxi Shi, Erin M. Poole, Joan O'Connell, Spero Manson, Luohua Jiang

**Affiliations:** ^1^ University of California Irvine, Irvine, CA, USA; ^2^ Joe C. Wen School of Population & Public Health, Henry and Susan Samueli College of Health Sciences, University of California, Irvine, Irvine, CA, USA; ^3^ University of Colorado Anschutz Medical Campus, Aurora, CO, USA

## Abstract

**Background:**

Relationships between comorbidities and cognitive impairment among American Indian and Alaska Native (AI/AN) adults remain unclear. This study explored the proportion of cognitive impairment and its association with various comorbidities in AI/AN communities across different age groups.

**Method:**

In 2019, a cross‐sectional survey was conducted among AI/AN volunteers aged 45+ residing in urban and rural areas across the Pacific Northwest, Rocky Mountains, and Northern Plains. Data on demographics, responses to culturally tailored Ascertain Dementia 8‐item Questionnaire (AD8), and self‐reported medical history of comorbidities were collected. Comorbidities included distress, diabetes, hypertension, heart disease, stroke, head injury, alcohol use disorder, and obesity. Cognitive impairment was defined as answering “Yes” to 2 or more AD8 questions, with the total number of “Yes” responses counted as the AD8 score. Higher AD8 scores indicate poorer cognitive function. Poisson regression was applied to model the associations of AD8 scores with demographics and comorbidities.

**Result:**

A total of 772 AI/AN participants were included, with a mean age of 65.0±8.2 years, and 70.3% were females. The overall proportion of cognitive impairment was 35.0%, with an unexpectedly high proportion observed in the youngest group aged 45‐60 (41.2%) (Table 1). In age and sex adjusted regression models, all the examined comorbidities were significantly associated with higher AD8 scores (Table 2). In fully adjusted models, distress [incidence rate ratios (IRR)=1.97] and diabetes (IRR=1.43) remained significantly associated with increased AD8 scores in both age groups. Head injury (IRR=2.20) and alcohol use disorder (IRR=1.33) presented significant associations with AD8 scores only in the <65 age group, while stroke (IRR=1.50) exhibited a significant association with increased AD8 scores only in the ≥65 age group (Figure 1).

**Conclusion:**

The proportion of cognitive impairment identified by AD8 in this AI/AN volunteer sample is high, especially among those aged 45‐60. Some associations between comorbidities and cognitive function differ by age groups. Early screening of cognitive impairment with convenient tools like AD8 questionnaires in middle‐age AI/AN adults is suggested, especially among those with head injury or alcohol use disorder.